# Diagnostic studies of abortion in Danish cattle 2015–2017

**DOI:** 10.1186/s13028-019-0499-4

**Published:** 2020-01-03

**Authors:** Godelind Alma Wolf-Jäckel, Mette Sif Hansen, Gitte Larsen, Elisabeth Holm, Jørgen Steen Agerholm, Tim Kåre Jensen

**Affiliations:** 1 National Veterinary Institute, Technical University of Denmark, Kemitorvet, 2800 Kongens Lyngby, Denmark; 2 Section for Veterinary Reproduction and Obstetrics, Department of Veterinary Clinical Sciences, Faculty of Health and Medical Sciences, University of Copenhagen, Højbakkegaard Alle 5A, 2630 Taastrup, Denmark; 3 Present Address: Section of Pathology, Department of Veterinary and Animal Sciences, University of Copenhagen, Ridebanevej 3, 1870 Frederiksberg C, Denmark

**Keywords:** Denmark, *Escherichia coli*, *Neospora caninum*, *Staphylococcus aureus*, *Trueperella pyogenes*, Zoonosis

## Abstract

**Background:**

Abortion is a major source of economic losses in cattle breeding. Abortion occurs due to a wide range of causes, but infections are the most frequently diagnosed. However, establishing an aetiological diagnosis remains challenging due to the large variety of bacteria, protozoa, viruses, and fungi that have been associated with abortion in cattle. Economic restraints limit the range of diagnostic methods available for routine diagnostics, and decomposition of the conceptus or lack of proper fetal and/or maternal samples further restrict the diagnostic success. In this study, we report recent diagnostic findings from bovine abortions in Denmark, a country that has a large dairy sector and is free from most infectious agents causing epizootic abortion in cattle. The aims of the study were: (i) to identify infectious causes of bovine abortion in Denmark, (ii) to categorise the diagnostic findings based on the level of diagnostic certainty, and (iii) to assess the diagnostic rate. Due to economic restraints, only a limited panel of routine diagnostic methods were available. Placentas and/or fetuses from mid- to late-term abortions and stillbirths (n = 162) were submitted to the Danish National Veterinary Institute between January 2015 and June 2017. The aborted materials were examined macroscopically, histologically, and by bacterial culture. Maternal blood samples were tested for bovine viral diarrhoea virus (BVDV) antibodies.

**Results:**

The likely aetiology of the abortion was diagnosed in 52 cases, resulting in a diagnostic rate of 33%. The most common cause was protozoal infection (19%) followed by infection with *Trueperella pyogenes* (3%), *Staphylococcus aureus* (2%), and non-haemolytic *Escherichia coli* (2%). Lesions in fetuses with a protozoal infection were consistent with neosporosis. In many cases (38%), inflammatory changes were found in the placenta and/or fetal organs but no specific aetiology was identified. Neither infection with *Brucella* spp. nor maternal BVDV antibodies were detected. The majority of submitting herds (92%) were each represented by fewer than three abortion cases over the study period.

**Conclusions:**

Protozoal infection, most likely neosporosis, was the most commonly diagnosed cause of abortion and the only one associated with potential epizootic abortion events. Despite using a reduced number of diagnostic methods in comparison to other abortion studies, the diagnostic rate of this study was within the range reported in an earlier Danish study, as well as in recent international studies. The low number of submitted cases per herd and the sparse anamnestic information provided at submission hampered conclusions on the potential epizootic character of the abortion events in question.

## Background

Good reproductive performance is of great importance to the economics of cattle breeding in both dairy and beef cattle herds. Sporadic abortions occur in all herds over time, but these are usually of minor importance and in many cases, the cause is not investigated. On the other hand, multiple abortions may occur suddenly or persist for a longer period and thus have an impact on the financial situation of the farm, e.g. due to lowered milk production, increased calving intervals, lack of sufficient future breeding stock, and fewer males for fattening or sale [1].

Causes of abortion may be either infectious or non-infections in aetiology. Although non-infectious causes have gained more attention during recent years, e.g. the identification of lethal haplotypes [e.g. 2, 3], infections are generally considered to be more important due to their significant abortifacient potential and because some organisms such as *Brucella* spp. are zoonotic. Furthermore, infections are traditionally more readily diagnosed than non-infectious causes [4–9].

Unlike many other countries, Denmark is in the fortunate position of being free from many important abortigenic pathogens. For example, an official eradication programme for bovine brucellosis was initiated in 1948, with the last case diagnosed in 1962 [10]. Since then, bovine herpesvirus type 1 (BHV-1), *Tritrichomonas foetus*, and *Campylobacter fetus* subsp. *venerealis* have also been eradicated [10]. Moreover, eradication of bovine viral diarrhoea virus (BVDV) is almost complete, with only one infected herd as of May 2019 [11]. In addition, some infections such as bovine leptospirosis are most likely to be extremely rare as they have never been recorded, and in the rare case that seropositive animals are identified, their sero-titers are low [12]. However, abortion is still of concern to Danish cattle breeders. As the last study on the causes of abortion in Danish cattle was published more than 20 years ago [6], we performed a diagnostic survey with the following aims: (i) to identify infectious causes of bovine abortion in Denmark, (ii) to categorise the diagnostic findings based on the level of diagnostic certainty, and (iii) to assess the diagnostic rate. Due to economic constraints, only a limited panel of routine diagnostic methods were available.

## Methods

### Animals

Aborted and stillborn bovine fetuses or fetal tissue, fetal placentas, and maternal blood samples submitted to the National Veterinary Institute of Denmark for diagnostics from January 2015 to June 2017 were prospectively included. Abortion was defined as expulsion of a non-viable fetus between gestation day 43 and 260, while stillbirth was defined as expulsion of the fetus after gestation day ≥ 260. Unless stated otherwise, both entities will be referred to as “abortion” in the following. Each submitted fetus or set of fetal organs (heart, lung, liver, spleen, and brain) counted as a single case, except for twins where both twins were submitted, which counted as one case. Cases in which only the placenta and/or a maternal blood sample were available were excluded from the study. Age determination of the fetus was based on the insemination and abortion dates, the insemination and/or expected calving dates provided by the submitting veterinarian, or the crown-rump-length (CRL) [13] in order of decreasing priority. In cases lacking these data, pulmonary histology was used to estimate the fetal age [14]. Abortions were categorised as having occurred during the first, second or third trimester of gestation, i.e. gestation months  ≤ 3, 4–6, and ≥ 7, respectively. Throughout the entire study period, the Danish Veterinary and Food Administration (DVFA) covered the routine diagnostic costs for up to five abortion cases per herd in order to encourage farmers to submit abortion cases and thereby increase the number of cases submitted for national brucellosis surveillance.

### Herd data

Information on insemination and abortion dates, breed of the dam, location of the farm, and the number of registered abortions in Denmark was retrieved from the Danish Cattle Database (SEGES P/S, Aarhus, Denmark).

### Necropsy and histopathological procedures

The fetuses and placentas were examined macroscopically and the CRL was measured. Specimens for histology were collected from the placenta, lung, liver, heart, and brain, fixed in 10% neutral buffered formalin, processed by routine methods, and embedded in paraffin [15, 16]. Tissue sections of 3–5 µm were cut and routinely stained with haematoxylin and eosin (H&E). Sections of placental specimens in which fungal infection was suspected were also stained with Grocott’s methenamine silver method.

### Bacteriological procedures

Liver, lung, and abomasal contents were cultured under aerobic conditions at 37 °C on Columbia agar plates supplemented with 5% calf blood (in house) as well as on Drigalski lactose agar plates (in house). The plates were inspected for bacterial growth after 16–20 h of cultivation and after an additional 24 h in cases of no or slow bacterial growth at first inspection. The selection of colonies for subculturing was based on colony morphology and the quantity of colony types that were not considered to be post-mortem contamination (e.g. *Proteus* spp.) based on the experience of the bacteriologist (GL). In cases of mixed bacterial growth, bacteria from the three most numerous and significant colony types were subcultured. Significance was assessed based on the presence of the same colony type in more than one organ culture per abortion case: the more organ cultures with the same colony type, the more significant the colony type. If more than three different colony types were present without any significant difference in their organ distribution, bacterial growth was described as unspecific mixed flora and subculturing was not performed. Colony material from the subcultures was analysed by matrix-assisted laser desorption/ionisation time-of-flight mass spectrometry (MALDI-TOF MS). Mass spectra were obtained using a microflex LT instrument (Bruker Daltonics, Bremen, Germany) calibrated using a bacterial test standard (Bruker Daltonics). The analyses were carried out in accordance with Bizzini et al. [17] using the MBT Compass software and the Biotyper MALDI-TOF MS database (Bruker Daltonics) supplemented with validated spectra from the National Veterinary Institute’s in-house database [18]. In order to screen for the presence of *Brucella* spp., specimens of fetal liver, lung, and spleen were pooled, homogenised, and cultured in 10% CO_2_ at 37 °C on selective agar plates containing horse serum, *Brucella* selective supplement (Oxoid A/S, Roskilde, Denmark), and ethanol. Growth of *Brucella* spp. was assessed after 48–72 h and after 7 and 10 days of incubation.

### Herd BVD status

The official BVD status at the time of submission ± 6 months was retrospectively assessed by searching the records of the National Veterinary Institute for herds that had submitted positive milk and/or blood samples during this period as part of the mandatory BVD surveillance programme.

### BVDV serology

Maternal serum was examined for the presence of antibodies against BVDV using a liquid-phase blocking ELISA [19].

### BVDV immunohistochemistry

Detection of the BVDV antigen in tissue sections of fetal cerebrum, cerebellum, and skin was carried out as described previously for formalin-fixed paraffin-embedded tissue [20]. Only cases originating from herds with a BVD positive status were analysed.

### Diagnostic criteria

The diagnostic findings were categorised into four main groups modified from Agerholm et al. [6].

#### Group 1. Likely cause of abortion identified

Diagnosis of bacterial infection was based on the isolation of bacteria and presence of lesions consistent with bacterial infection. Mycosis was diagnosed by the presence of hyphae or yeast cells in tissue sections associated with inflammation. Protozoal abortion was diagnosed by findings of non-suppurative inflammation in fetal organs as follows: cases were considered positive if focal to multifocal non-suppurative necrotising encephalitis was found together with non-suppurative interstitial myocarditis and/or non-suppurative hepatitis. In the absence of brain lesions or exclusion of the brain due to extensive decomposition, the presence of non-suppurative interstitial myocarditis together with non-suppurative hepatitis was regarded as being diagnostic of protozoal abortion. Infection with BVDV was diagnosed by demonstration of the BVDV antigen within fetal tissues.

In the case of dual infection (i.e. bacterial and protozoal infections), the case was diagnosed as protozoal abortion because the protozoa-associated inflammatory lesions were more severe than the bacteria-associated lesions.

#### Group 2. Lesions present, specific aetiology not identified

This group included cases for which a specific cause was not detected but where lesions were present in the fetus and/or the fetal placenta. Cases were categorised based on the most severe lesion observed.

#### Group 3. Bacteria isolated from at least two organs, lesions not found

This group consisted of cases from which opportunistic pathogenic bacteria were isolated from at least two organs, but without associated lesions. Isolation of *Proteus* spp. was regarded as contamination.

#### Group 4. No likely cause identified

This group comprised cases without fetal or placental lesions, including cases where bacteria were isolated from only one organ without concomitant lesions.

### Epizootic criteria

Herds with at least two submitted cases were included in this analysis and only cases with an established aetiology were considered (Group 1). Two approaches were then used to identify potential epizootic abortion events, i.e. multiple abortions occurring within one herd due to the same agent and at a frequency higher than that expected during the given time period. Firstly, herds with quantifiable anamnestic information with a frequency of  ≥ 4 abortions in one week,  ≥ 6 abortions in one month or  ≥ 12 abortions in 3 months, regardless of the size of the herd (adapted from Ancker and Agerholm [21]) were identified as having potential epizootic abortion. Anamnestic information was defined as data on the current abortion frequency in the herd provided by the veterinarian upon submission of the abortion case. Since quantifiable anamnestic information was sparse, a second approach was used: all herds from which ≥ 2 abortion cases were submitted within 21 days of each other were identified as having potential epizootic abortion. For both approaches, an epizootic event was diagnosed in the identified herds if the diagnosed aetiology included a pathogen known to cause epizootic abortion, and if ≥ 2 cases per herd were diagnosed with the same aetiology.

## Results

A total of 162 cases comprising 153 abortion cases and nine cases of stillbirth were included in the study. The abortion cases consisted of 138 singletons and 26 twin fetuses representing 15 cases of abortion of twins. In most cases of abortion (n = 152), an intact fetus was submitted, while only the head and fetal organs were submitted for one case. All stillborn calves were singletons and were submitted as intact carcasses except for in one case where only the head and internal organs were submitted.

**Fig. 1:**
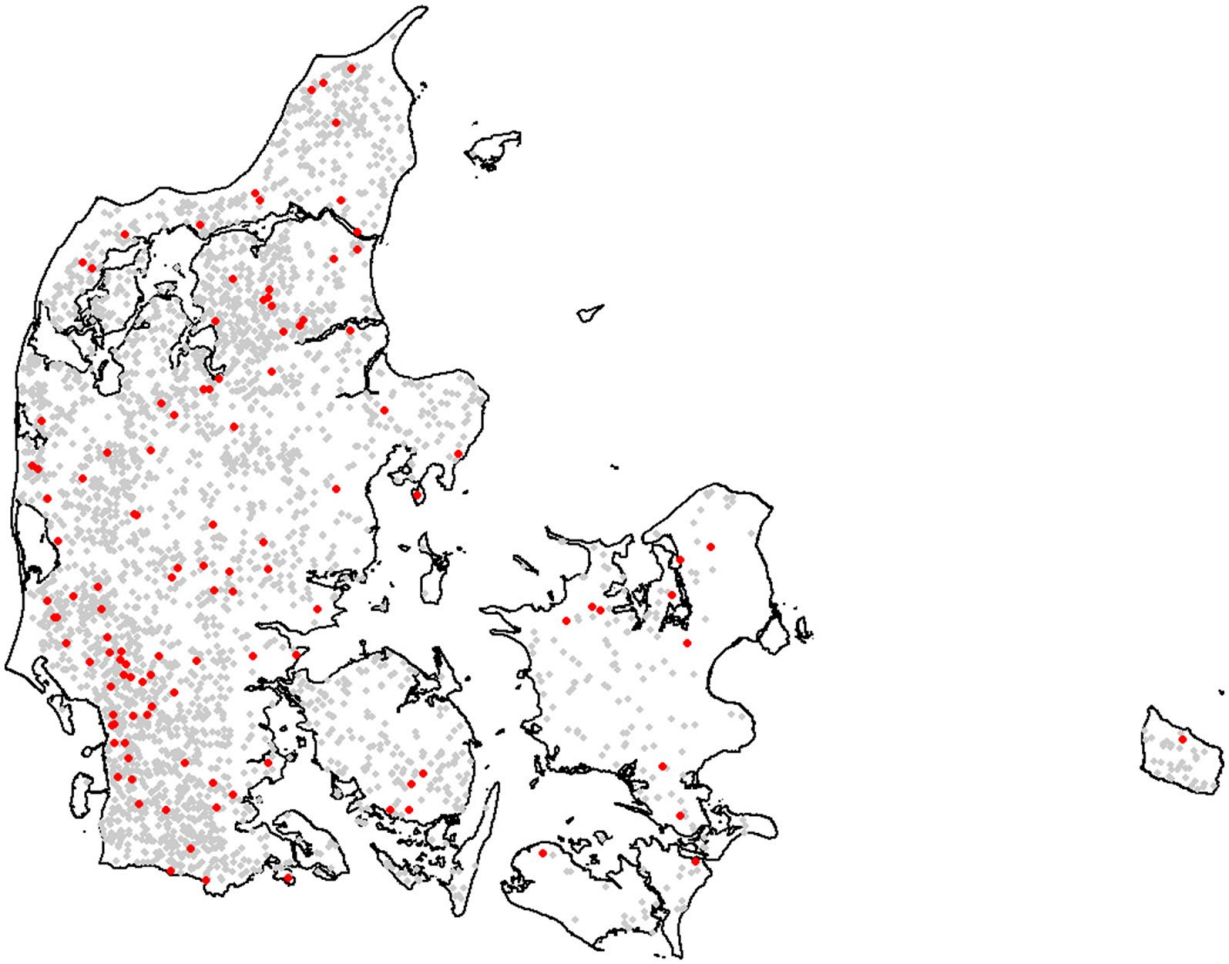
Geographical distribution of Danish farms submitting bovine abortion cases for diagnostics from January 2015 until June 2017. Submitting farms are depicted as red dots. All Danish dairy farms registered at the end of 2017 are depicted as grey dots. Abortion cases were submitted from all parts of Denmark, and areas of high dairy farm density were represented by a relatively higher number of submitting farms

The majority of the fetal membranes and internal organs submitted were histologically assessable (Table 1). Maternal blood samples were submitted in 145 cases—137 from abortion cases and eight from stillbirth cases. The cases originated from 116 herds, with 92% of the herds being represented by one or two cases (Table 2). Most fetuses (67%) were aborted during the last trimester, while 33% were aborted during the second trimester and one during the first trimester. Nine of the cases aborted during the last trimester were stillbirths. The breed distribution of aborting cows was as follows: 62% Danish Holstein followed by 13% Danish Jersey, 9% mixed breeds, 7% Danish Red, and 9% other, thereby closely mirroring the breed distribution among the Danish dairy cow population [22]. The majority of the cases originated from dairy farms (94%) and 91% of all submitting farms were conventional, i.e. non-organic farms. Submissions originated from all parts of Denmark, and areas of high dairy farm density were represented by a large number of submitting farms (Fig. 1). The investigated cases represented 0.8% of the abortions registered in the Danish Cattle Database during the study period.

**Table 1:** The number of abortion cases for which fetal placenta and organs were submitted and the number of tissues with histological lesions

Number	Placenta	Lung	Liver	Heart	Brain
Histologically assessable^a^	123	148	132	156	157
Excluded from histology	8	12	29	3	3
Total submitted/sampled	131	160	161	159	160
Lesions present	78	46	35	37	32

^a^Defined as suitable for assessing the presence/absence of cellular inflammation

**Table 2:** Number of bovine abortion submissions per herd

Cases per herd	Number of herds	% of submitting herds	Number of cases	% of total cases
1–2	107	92.2	127	78.4
3–5	8	6.9	28	17.3
7	1	0.9	7	4.3
Total	116	100	162	100

**Fig. 2:**
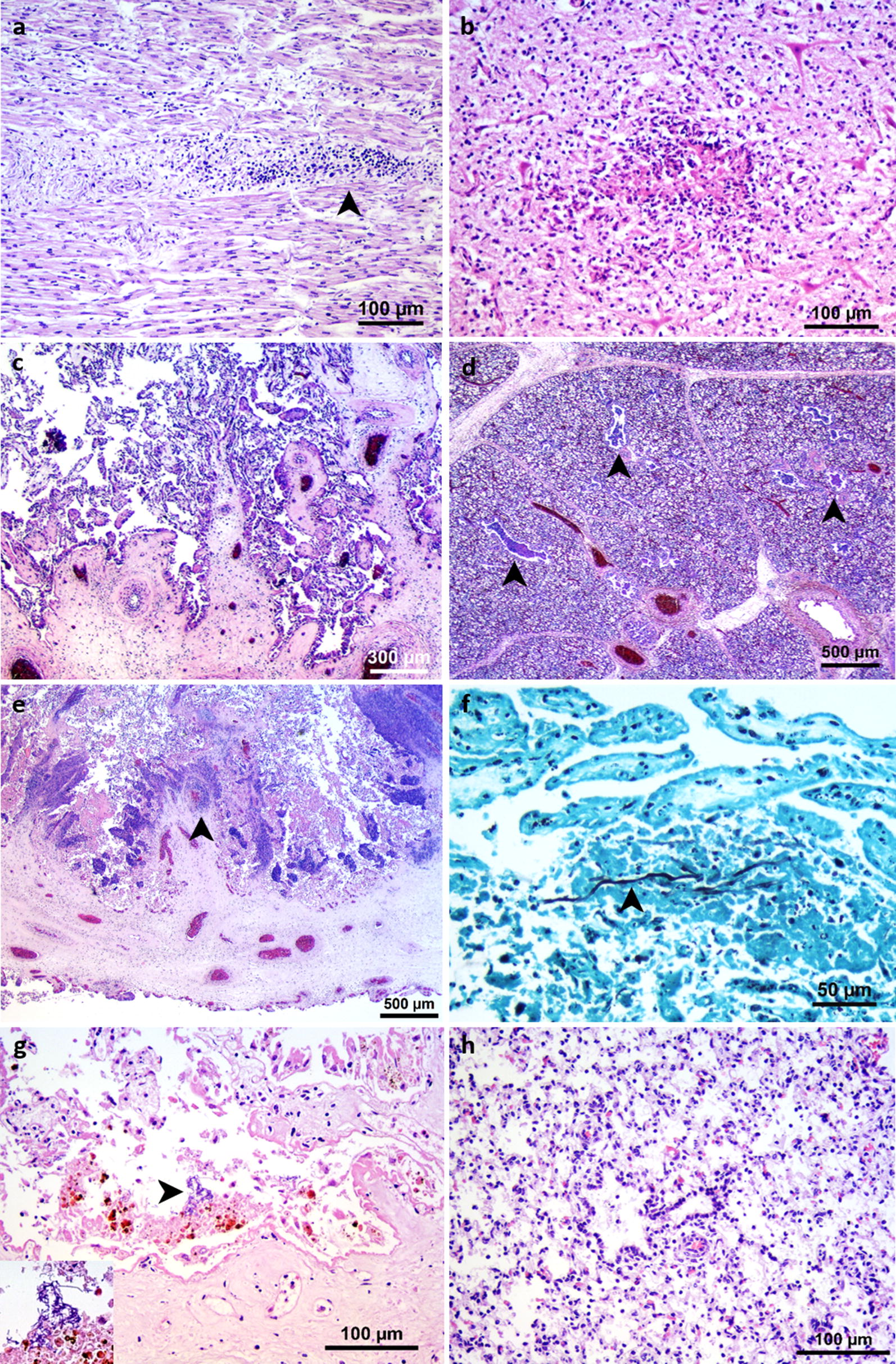
Histological findings from bovine abortion cases in which an aetiological diagnosis was established (Group 1). (a) Mononuclear interstitial myocarditis (arrowhead) and (b) necrotic focus in cerebellar grey matter in a case of protozoal abortion. (c) Necrosuppurative placentitis and (d) suppurative bronchopneumonia with numerous bronchioles filled with cellular exudate and bacterial micro colonies (arrowhead) in a case of abortion due to *Trueperella pyogenes* infection. (e) Necrotising placentitis with vasculitis (arrowhead) and (f) intralesional septate fungal hyphae (arrowhead) in a case of abortion due to fungal infection. (g) Necrosuppurative placentitis with intralesional filamentous bacteria (*arrowhead*, inset) and (h) suppurative bronchopneumonia in a case of abortion due to *Bacillus licheniformis* infection. **a**–**e**, **g**, **h** H&E, **f** Grocott’s methenamine silver

A specific cause of abortion was diagnosed in 53 cases (Group 1, Table 3). Protozoal abortion was diagnosed in 31 cases originating from 21 herds. Non-suppurative interstitial myocarditis (Fig. 2a) was the most consistent lesion detected (all 31 cases), followed by non-suppurative hepatitis (26 cases), and focal necrotising encephalitis (23 cases, Fig. 2b). These lesions are consistent with neosporosis, but a specific diagnostic method was not used to confirm this. The distribution of protozoal abortion cases per herd was as follows: 16 herds with one case, three herds with two cases, one herd with four cases, and one herd with five cases. In one of the cases originating from a herd that submitted only one protozoal abortion, concomitant infection with *Lactococcus garvieae* was found. The diagnosis was based on the presence of non-suppurative encephalitis, myocarditis, and hepatitis together with necrosuppurative placentitis and suppurative bronchopneumonia, and the isolation of *L. garvieae* from lung, liver, and abomasal contents. Abortion caused by bacteria was diagnosed in 19 cases. *Trueperella pyogenes* was the most frequently isolated species followed by non-haemolytic *Escherichia coli*, *Staphylococcus aureus,* and *Listeria monocytogenes* (Table 3). *Bacillus licheniformis*, *Streptococcus* spp., *Klebsiella pneumoniae*, *Aeromonas* spp., and *L. garvieae* were diagnosed as abortifacient in one case each. Suppurative to necrosuppurative placentitis was the most prevalent histological finding among the bacterial abortions. Concomitant suppurative bronchopneumonia was found in six cases. Mycosis was diagnosed in two cases originating from two herds, and fungal structures were identified within the placental lesions in both cases using Grocott’s methenamine silver stain. In one case, fungal hyphae were associated with necrosis of chorionic villi and vasculitis within the chorionic plate. In the other case, spherical yeast cells were associated with necrosis of chorionic villi as well as with cellular exudate within the bronchiolar and alveolar lumen of the lung. Representative lesions associated with bacterial and fungal infections are shown in Fig. 2c–f. All maternal blood samples were negative for BVDV antibodies. Two out of the 116 submitting herds had a BVD-positive status during the study period ± 6 months. Both positive herds were represented in the study by one abortion case each. BVDV was diagnosed as the cause of abortion in one of these cases based on the detection of the BVDV antigen in the fetal cerebrum and cerebellum. The corresponding dam had been seropositive for BVDV 6 months prior to abortion, but a maternal blood sample was not submitted at abortion. The brain and skin samples of the other case were immunohistochemically negative and the corresponding maternal blood sample was seronegative at abortion. Organ pools of all fetuses were culture negative for *Brucella* spp.

**Table 3:** Diagnostic findings in 162 bovine abortions and stillbirths originating from 116 Danish herds

Diagnostic group	Number of cases	% of total
1. Likely cause of abortion identified	53^a^	33^a^
*Neospora caninum*	31	19
*Trueperella pyogenes*	5	3
*Escherichia coli,* non-haemolytic	4	2
*Staphylococcus aureus*	3	2
*Listeria monocytogenes*	2	1
Other bacterial species^b^	5	3
Fungi	2	1
Bovine viral diarrhoea virus	1	< 1
2. Lesions present, specific aetiology not identified	62^a^	38^a^
Suppurative to necrosuppurative placentitis and suppurative bronchopneumonia	12	7
Suppurative to necrosuppurative placentitis	26	16
Suppurative bronchopneumonia	14	9
Non-suppurative placentitis	8	5
Interstitial pneumonia	1	< 1
Granulomatous pneumonia	1	< 1
3. Bacteria isolated^c^, lesions not found	15^a^	9
*Escherichia coli*, non-haemolytic	5	3
*Acinetobacter* spp.	4	2
*Acinetobacter* spp. and *Aerococcus* spp.	2	1
*Aerococcus* spp.	1	< 1
*Hafnia alvei*	1	< 1
*Lactobacillus curvatus*	1	< 1
*Vagococcus fluvialis*	1	< 1
4. No likely cause identified	32^a^	20^a^
Total	162	100

^a^Accumulated figures within the respective diagnostic group

^b^*Bacillus licheniformis*, *Streptococcus* spp., *Klebsiella pneumoniae*, *Aeromonas* spp., *Lactococcus garvieae* (one case each)

^c^Isolated from at least two specimens per case

In 62 cases, inflammation was detected in the placenta and/or fetal organs without an aetiology being identified (Group 2, Table 3). Suppurative inflammation consistent with bacterial infection was detected in 52 cases. In 12 of those cases, both placentitis and bronchopneumonia were found, while placentitis alone was observed in 26 cases, and bronchopneumonia was observed 14 cases. Non-suppurative placentitis was the most severe lesion in eight cases, while granulomatous bronchopneumonia and interstitial pneumonia were found in one case each.

In 15 cases, the same bacterial genus or species were isolated from at least two specimens per case, yet no lesions were found in the placenta and/or fetal organs (Group 3, Table 3). *Acinetobacter* spp. (six cases), non-haemolytic *E. coli* (five cases), and *Aerococcus* spp. (three cases) were the most frequently isolated bacteria. Non-haemolytic *E. coli* and *Hafnia alvei* were isolated as pure cultures from the abomasal contents in two cases and one case, respectively.

In 32 cases, no likely cause of abortion was diagnosed due to the absence of lesions and because all cultured specimens were sterile (eight cases), a non-specific mixed flora was cultured (16 cases), or because bacteria were cultured from only one specimen (six cases; Group 4, Table 3). In the remaining two cases, more than one bacterial species was isolated, but each species came from only one specimen per case. In one of these two cases, *B. licheniformis* and *L. monocytogenes* were isolated as part of a mixed flora from the liver and abomasal contents, respectively. The thorax of this fetus had been opened before submission and the heart and lung were missing. Post-mortem degradation of the fetal organs was noted and interpreted as having occurred at least partially after expulsion of the fetus. In the other case, *Staphylococcus sciuri* and non-haemolytic *E. coli* were cultured as part of a mixed flora from the liver and abomasal contents, respectively.

Protozoal abortion was diagnosed in up to five cases per herd, while abortion due to bacterial or fungal infections occurred in only one case per herd (Table 4). Based on quantifiable anamnestic information, two herds with two cases each were identified with a potential epizootic abortion event. Protozoal infection was the cause in both cases from one herd, while *T. pyogenes*, and *S. aureus* were the abortifacients in one case each from the other herd. Based on the criterion of submitting two or more abortion cases within 21 days, five herds with a total of 13 cases were identified as having had a potential epizootic abortion event. Protozoal infection was identified as the cause in all cases from four of those herds, with three herds being represented by two cases and one herd by five cases. The fifth herd was the same one identified based on quantifiable anamnestic information and with *T. pyogenes* and *S. aureus* as aetiologies. An epizootic abortion event represented by those two cases was regarded as unlikely because *T. pyogenes* and *S. aureus* are only known to cause sporadic abortion [16]. The herd with five protozoal abortion cases was the same one identified based on quantifiable anamnestic information, however, the first three cases from this herd had been submitted without anamnestic information on abortion rate and were therefore not identified by the anamnestic information-based approach. As a result, potential epizootic abortion events were identified in a total of four herds, and protozoal infection was identified as the cause in all of these herds. The fetuses were aborted in the second and third trimester, during first to fifth parity, and from dams between 2 and 6 years of age.

**Table 4:** Correlation between the number of submitted cases per herd and the number of cases diagnosed with a certain aetiology

Number of cases per herd	Number of submitted cases per herd						Total number of herds
	1	2	3	4	5	7	
Protozoal abortion (n = 1)	13	2	1	0	0	0	16
Protozoal abortion (n = 2)	NA	3	0	0	0	0	3
Protozoal abortion (n = 4)	NA	NA	NA	0	0	1	1
Protozoal abortion (n = 5)	NA	NA	NA	NA	1	0	1
*Trueperella pyogene*s (n = 1)	2	2	1	0	0	0	5
*Escherichia coli* (n = 1)	1	1	2	0	0	0	4
*Staphylococcus aureus* (n = 1)	2	1	0	0	0	0	3
*Listeria monocytogenes* (n = 1)	2	0	0	0	0	0	2
*Bacillus licheniformis* (n = 1)	0	1	0	0	0	0	1
Mycosis (n = 1)	1	1	0	0	0	0	2
Bovine viral diarrhoea virus (n = 1)	1	0	0	0	0	0	1

Inflammation was detected in 11 out of 15 abortions of twins: protozoal abortion was diagnosed in two cases, while fungal infection was the cause in one case. In the remaining eight cases with inflammatory lesions, no likely aetiology of abortion was established. Lesions consistent with placental and/or fetal infection were detected in seven out of nine stillbirths. Protozoal infection was diagnosed in two of these cases, non-haemolytic *E. coli* in one case, and lesions consistent with bacterial infection were found in four cases, though without identification of the bacterial aetiology. In the remaining two cases, no lesions were found and there was no anamnesis of dystocia.

## Discussion

In this study, an infectious aetiology was established in 33% of the examined cases. Protozoal infection was the most prevalent cause and the only one that could be associated with potential epizootic abortion events. Several major abortifacient pathogens have been eradicated from Denmark. The last case of BHV-1 was recorded in 2005, *Tritrichomonas foetus* in 1990, *Brucella abortus* in 1962, and *Campylobacter fetus* subsp. *venerealis* in 1995 [10]. Furthermore, a specific examination was performed to confirm the absence of bovine brucellosis in Denmark following demands from the European Union. No specific analyses were performed for other pathogens considered by the DVFA to be absent in Denmark. Strict surveillance rules are in place concerning venereally transmitted diseases: breeding bulls are tested serologically for *B. abortus* and BHV-1 infection, and for *C. fetus* subsp. *venerealis* and *T. foetus* infection via culture of preputial wash samples. As the majority of Danish dairy cows are bred via artificial insemination using certified bulls, and as most of the submitted cases in this study (94%) originated from dairy herds, it is fair to assume that the submitted fetuses were free from these bacterial infections, although they were not specifically tested for any of these diseases except for brucellosis. BHV-1 surveillance of cows is also mandatory and is done by either testing milk from dairy herds or blood from beef cattle. No positive herds have been found in Denmark since 2005. We therefore assumed that the submitted fetuses were also free from BHV-1. Surveillance for venereal diseases is targeted at bulls used for artificial breeding. Females are not tested unless the herd veterinarian suspects such a disease. Therefore, it cannot be ruled out completely that these infections are present in some females, but it is difficult for diseases spread by venereal transmission to persist in a herd without natural breeding. Even though specific analyses were not performed for all of these agents in the present study, their presence in an abortion case would have been suspected based on characteristic lesions, and in such cases, targeted diagnostics would have been applied. It is therefore reasonable to consider that the submitted fetuses were free of these infections.

An eradication programme for BVD has been in place in Denmark since 1994. BVD was almost eradiated in 2006, but a small number of herds still test positive each year [10]. Only two of the fetuses in our study were tested for the BVDV antigen and BVDV could therefore have been overlooked as a cause of abortion. However, maternal blood samples from 145 out of 162 dams were analysed for BVDV antibodies and found to be seronegative. It is possible that some fetuses could have been aborted during the acute stage of a BVDV infection. To rule this out, herd data covering the study period ± 6 months from the national surveillance based on quarterly bulk tank milk analyses (dairy cattle) or blood sampled at slaughter (beef cattle) were retrospectively assessed. All abortions (n = 2) originating from the two herds with a BVD-positive status were retrospectively analysed for the presence of the BVDV antigen and one of them was found to be BVDV positive. Even though the majority of cases in the present study were not directly tested for BVDV, the abovementioned supportive data allow us to conclude that BVDV played a negligible role as abortifacient agent in our study population.

The diagnostic rate of bovine abortion usually lies below 50% [4, 6, 8, 9, 23]. The diagnostic rate in our study was 33%, which is within the normal range of most diagnostic studies, but lower than a recent study from Australia and New Zealand in which a broad range of serological and immunohistochemical methods, in addition to histology and bacterial culture, were applied [7]. An increase in the diagnostic rate over time has been reported in studies from the USA—from 33% in the 1990s in South Dakota [23] to over 44% in the 2000s in California [9], and 57% in 2016 in California [8]. The prevalence of infectious abortifacients in a sampled population can have a significant influence on the diagnostic rate. High diagnostic rates are often reported in geographical regions with a high prevalence of known abortifacients, e.g. the study from California [8] reported a diagnostic rate of 57% and stated that *Pajaroellobacter abortibovis* (causing enzootic bovine abortion) and *Neospora caninum* have a high local prevalence and are readily diagnosed with the methods established at the local diagnostic laboratory. The diagnostic rate in our study (33%) was similar to that previously reported in Denmark in 1997 (35%) [6]. The diagnostic criteria for these two studies were similar, but differed slightly for Group 1: firstly, BVDV infection was almost exclusively assessed by serological analysis of maternal serum and surveillance data in our study, while Agerholm et al. [6] based their diagnoses on fetal virus demonstration or on the detection of fetal antibodies. Secondly, protozoal abortion (neosporosis) was diagnosed only on the basis of histological findings in H&E stained sections in our study, while Agerholm et al. [6] confirmed cases with suspicious microscopic lesions by immunohistochemistry and/or fetal serology. In our study, the diagnosis of ‘protozoal abortion’ was entirely based on the observation of focal to multifocal non-suppurative necrotising encephalitis together with non-suppurative interstitial myocarditis and/or non-suppurative hepatitis. Even though such lesions are not pathognomonic for fetal neosporosis, but may be associated with other protozoal infections such as fetal sarcocystosis and toxoplasmosis [16], we assumed that *N. caninum* was the most probable aetiology in all cases of protozoal abortion: neosporosis is by far the most common cause of protozoal bovine abortion compared to e.g. sarcocystosis or toxoplasmosis [24, 25]. *Sarcocystis* spp. and *Toxoplasma gondii* cysts are more readily detected by histology than *N. caninum* cysts, and were not observed in our study. However, immunohistochemistry or another agent-specific methodology would have been a superior diagnostic method compared to histopathology.

The observed prevalence of *N. caninum*-associated abortion (19%) is within the normal range of prevalence recorded in Denmark between 1995 and 2017 (12% to 25%, Table 5) [12] and worldwide [26]. Histopathology is required to diagnose *N. caninum*-associated abortion because lesions must be identified in order to differentiate between latent and abortigenic infections [27]. The serological status of the dam and/or the fetus was not determined in this study because it was not considered to provide definitive evidence regarding the cause of abortion [28, 29]. The serological status of the fetus does not necessarily reflect the true status of fetal infection with *N. caninum*: infection of the fetus before gestation day 100 is unlikely to result in fetal antibody production due to an immature immune response, while exposure of an immunocompetent fetus induces a humoral immune response [29]. Infection before the foetus is immunocompetent is more likely to result in abortion than an infection at a later stage [29], and can result in the abortion of seronegative fetuses with typical protozoal lesions [28]. Fetuses infected and aborted after becoming immunocompetent will be seropositive if not aborted during the acute stage of infection [26, 30]. Furthermore, serological examination of the dam is only indicative of maternal exposure to *N. caninum* and does not reflect whether the parasite has been transmitted to the fetus during the current pregnancy, nor if the parasite has been the cause of abortion [27, 31].

**Table 5:** Summary of diagnostic findings in bovine abortion cases from Denmark investigated at the National Veterinary Institute between 1995 and 2017

Cause of abortion	Study [5] % of total	This study % of total	Bovine abortions investigated at the National Veterinary Institute, Denmark [11] % of total^a^						
			1996	1997	1998	1999	2000	2001	2014^g^
Diagnostic group 1^b^	35	33	43	34	42	38	43	38	33
Bovine viral diarrhoea virus	13	< 1	7	7	4	1	1	0	1
Protozoal abortion^c^	10^d^	19^e^	16^f^	15^f^	21^f^	25^f^	16^f^	17^f^	10^d^
*Trueperella pyogenes*	2	3	8	3	6	5	7	2	6
*Bacillus licheniformis*	4	NA	5	2	3	1	1	1	2
*Staphylococcus aureus*	NA	2	< 1	1	2	1	3	3	NA
*Listeria monocytogenes*	< 1	1	< 1	1	2	0	0	0	2
Other bacterial species	1	3	3	1	1	1	6	5	11
Fungi	5	1	2	3	3	4	9	10	1
Diagnostic group 2^b^	12	38	8	8	10	7	8	15	42
Diagnostic group 3^b^	6	9	9	3	4	11	1	8	NA
Diagnostic group 4^b^	46	20	40	55	45	44	48	39	21
Total investigated cases	186	162	233	189	192	169	150	89	139

^a^Data for 1995 and 2015–2017 are from [5] and the current study, respectively

^b^Accumulated figures within the respective diagnostic group

^c^Diagnostics for neosporosis began at the end of 1994

^d^Histologically identified cases confirmed by immunofluorescence and/or maternal serology

^e^Diagnosis based on histological findings from H&E stained tissue sections

^f^Combination of histological findings from H&E stained tissue sections and immunofluorescence used to establish diagnosis

^g^November 2013 to December 2014

In our study, aborted materials were submitted at the discretion of the farmer or the responsible veterinarian from all of Denmark over a period of 2.5 years. *N. caninum* was identified as the only potential cause of epizootic abortion events. The approach based on the submission time point identified more potential epizootic abortion events than the anamnesis-based approach, and included all of the events identified by the latter. Since the DVFA covered the routine diagnostic costs for up to five abortion cases per herd, the motivation to submit sporadically occurring abortion cases might have been higher than if the analyses had been at the farmers’ expense. Cases representing epizootic abortion events might therefore be underrepresented in the study.

Twinning in cattle is associated with a significantly increased risk of abortion if the twin pregnancy is unilateral, while bilateral twin pregnancy does not increase the abortion risk [32]. Unilateral twin pregnancy constitutes around 56% of the twin pregnancies in Holsteins [32] and represents a non-infectious cause of abortion [32]. Lesions consistent with infection of the placenta and/or the fetus were found in the majority of twin abortions in this study (11 out of 15 cases), suggesting that infection was the cause of abortion rather than twinning itself. Our findings emphasise the diagnostic value of investigating most twin abortions for infectious causes, though bearing in mind that abortion due to a unilateral twin pregnancy cannot be diagnosed post mortem.

Inflammation mainly suggestive of bacterial infection but without identification of the agent was found in 38% of cases, thereby representing the largest diagnostic group. Applying a broader panel of diagnostic methods, e.g. including anaerobic bacterial culture, culturing placental specimens, and using culture-independent methods might have increased the number of cases with a specific bacterial aetiology.

Cases presented to a diagnostic laboratory are by definition a convenience sample [8] and do not necessarily represent the true fetal loss in the sample population. Our data show that the Danish dairy cow population was reflected well by the study population in terms of geographical distribution, breed distribution, and production type. However, the number of submitted abortion cases constituted less than 1% of the abortions registered during the study period, indicating that a sample consisting of > 150 fetuses is still very limited.

When comparing our study with the previous Danish study [6], we noted a similar diagnostic rate and distribution of aetiological agents (with the exception of the almost eradicated BVDV infection), despite our study being characterised by a reduced number of diagnostic methods and sampling over a longer period of time.

## Conclusions

Protozoal infection, most likely representing neosporosis, was the most commonly diagnosed cause of abortion and the only one associated with potential epizootic abortion events. Despite using fewer diagnostic methods compared to other abortion studies, the diagnostic rate of this study was within the normal range of rates reported by an earlier Danish study, as well as by recent international studies. The low number of submitted cases per herd together with sparse anamnestic information provided at submission made it difficult to draw conclusions about the potential epizootic character of the abortion events in question.

## Data Availability

The datasets used and/or analysed in the current study are available from the corresponding author upon reasonable request.

## Abbreviations

BVD: bovine viral diarrhoea
BVDV: bovine viral diarrhoea virus
BHV-1: bovine herpesvirus type 1
CRL: crown rump length
DVFA: Danish Veterinary and Food Administration
ELISA: enzyme-linked immunosorbent assay
H&E: haematoxylin and eosin staining
MALDI TOF MS: matrix-assisted laser desorption/ionisation time-of-flight mass spectrometry

## References

[1] De Vries A (2006). Economic value of pregnancy in dairy cattle. J Dairy Sci..

[2] Charlier C, Li W, Harland C, Littlejohn M, Coppieters W, Creagh F (2016). NGS-based reverse genetic screen for common embryonic lethal mutations compromising fertility in livestock. Genome Res..

[3] Adams HA, Sonstegard TS, VanRaden PM, Null DJ, Van Tassell CP, Larkin DM (2016). Identification of a nonsense mutation in APAF1 that is likely causal for a decrease in reproductive efficiency in Holstein dairy cattle. J Dairy Sci..

[4] Syrjälä P, Anttila M, Dillard K, Fossi M, Collin K, Nylund M (2007). Causes of bovine abortion, stillbirth and neonatal death in Finland 1999–2006. Acta Vet Scand..

[5] Carpenter TE, Chrièl M, Andersen MM, Wulfson L, Jensen AM, Houe H (2006). An epidemiologic study of late-term abortions in dairy cattle in Denmark, July 2000–August 2003. Prev Vet Med..

[6] Agerholm JS, Willadsen CM, Nielsen TK, Giese SB, Holm E, Jensen L (1997). Diagnostic studies of abortion in Danish dairy herds. J Vet Med Ser A..

[7] Reichel MP, Wahl LC, Hill FI (2018). Review of diagnostic procedures and approaches to infectious causes of reproductive failures of cattle in Australia and New Zealand. Front Vet Sci..

[8] Clothier K, Anderson M (2016). Evaluation of bovine abortion cases and tissue suitability for identification of infectious agents in California diagnostic laboratory cases from 2007 to 2012. Theriogenology.

[9] Anderson ML (2007). Infectious causes of bovine abortion during mid- to late-gestation. Theriogenology.

[10] Danish Veterinary and Food Administration. Annual report on animal health in Denmark 2017. 2018. https://www.foedevarestyrelsen.dk/english/Animal/AnimalHealth/Pages/default.aspx. Accessed 2 June 2019.

[11] Landbrug og Fødevarer - SEGES [Danish Agriculture and Food Council]. KvægVet historik for BVD [Cattle Vet history for BVD]. https://kvaegvet.dk/BVD/AABvdHist.html. Accessed 7 May 2019.

[12] National Veterinary Institute. Kvægaborter – diagnostiske undersøgelser 1992–2001 og 2014–2017 [Results of diagnostic investigations in bovine abortions 1992–2001 and 2014–2017]. Annual reports on veterinary diagnostic investigations at the National Veterinary Institute, 1992–2001 and 2014–2017. Frederiksberg C, Denmark: Technical University of Denmark (DTU). Annual report ISBN 87–987164–1–7.

[13] University of Wisconsin. Bovine crown-rump-length calculator. https://www.ansci.wisc.edu/jjp1/ansci_repro/lab/lab12_03/crown-rump_analysis3.html. Accessed 15 Aug 2018.

[14] Schnorr B. Entwicklung der Atmungsorgane [Development of the respiratory system]. Embryol der Haustiere. 4th ed. Ferdinand Enke; 1989.

[15] Taylor RF, Njaa BL, Njaa BL (2012). General approach to fetal and neonatal loss. Kirkbride’s diagnosis of abortion and neonatal loss in animals.

[16] Anderson ML, Njaa BL (2012). Disorders of cattle. Kirkbride’s diagnosis of abortion and neonatal loss in animals.

[17] Bizzini A, Durussel C, Bille J, Greub G, Prod’hom G (2010). Performance of matrix-assisted laser desorption ionization-time of flight mass spectrometry for identification of bacterial strains routinely isolated in a clinical microbiology laboratory. J Clin Microbiol.

[18] Nonnemann B, Lyhs U, Svennesen L, Kristensen KA, Klaas IC, Pedersen K (2019). Bovine mastitis bacteria resolved by MALDI-TOF mass spectrometry. J Dairy Sci..

[19] Rønsholt L, Nylin B, Bitsch V. A BVDV antigen- and antibody blocking ELISA (DVIV) system used in a Danish voluntary eradication program. In: Edwards S, Paton D, Weensvort G, editors. In: Proc third ESVV Symp perstivirus infect. Lelystad, The Netherlands: Weybridge: Central Veterinary Laboratory, European Society for Veterinary Virology; 1997. p. 150–3.

[20] Hilbe M, Stalder H, Peterhans E, Haessig M, Nussbaumer M, Egli C (2007). Comparison of five diagnostic methods for detecting bovine viral diarrhea virus infection in calves. J Vet Diagn Invest..

[21] Ancker S, Agerholm J. Abortmanual - Vejledning i håndtering af abortproblemer [Abortion manual—guidance on handling abortion problems]. 2010. https://docplayer.dk/8056845-Abortmanual-vejledning-i-haandtering-af-abortproblemer-2-udgave.html. Accessed 5 Oct 2016.

[22] Landbrug og Fødevarer [Danish Agriculture and Food Council]. Kvæg [Cattle]. https://lf.dk/viden-om/landbrugsproduktion/husdyr/kvag. Accessed 15 Aug 2018.

[23] Kirkbride CA (1992). Etiologic agents detected in a 10-year study of bovine abortions and stillbirths. J Vet Diagnostic Investig..

[24] Canada N, Meireles CS, Rocha A, da Costa JM, Erickson MW, Dubey JP (2002). Isolation of viable *Toxoplasma gondii* from naturally infected aborted bovine fetuses. J Parasitol..

[25] Conrad PA, Barr BC, Sverlow KW, Anderson M, Daft B, Kinde H (1993). In vitro isolation and characterization of a *Neospora* sp. from aborted bovine foetuses. Parasitology.

[26] Dubey JP, Schares G, Ortega-Mora LM (2007). Epidemiology and control of neosporosis and *Neospora caninum*. Clin Microbiol Rev..

[27] Dubey JP, Schares G (2006). Diagnosis of bovine neosporosis. Vet Parasitol..

[28] Sörgel SC, Müller M, Chares G, Grossmann E, Neuss T, Puchta H (2009). Beteiligung von *Neospora caninum* bei Rinderaborten in Nordbayern [Contribution of *Neospora caninum* to bovine abortions in Northern Bavaria]. Tierarztl Umsch.

[29] Dubey JP, Buxton D, Wouda W (2006). Pathogenesis of bovine neosporosis. J Comp Pathol..

[30] Andrianarivo AG, Barr BC, Anderson ML, Rowe JD, Packham AE, Sverlow KW (2001). Immune responses in pregnant cattle and bovine fetuses following experimental infection with *Neospora caninum*. Parasitol Res..

[31] Dubey JP (2003). Review of *Neospora caninum* and neosporosis in animals. Korean J Parasitol..

[32] Garcia-Ispierto I, López-Gatius F (2019). Abortion in dairy cattle with advanced twin pregnancies: Incidence and timing. Reprod Domest Anim..

